# Formulation, Characterisation, and in Vitro Skin Diffusion of Nanostructured Lipid Carriers for Deoxyarbutin Compared to a Nanoemulsion and Conventional Cream

**DOI:** 10.3390/scipharm84040634

**Published:** 2016-07-20

**Authors:** Rendra P. Tofani, Yeyet C. Sumirtapura, Sasanti T. Darijanto

**Affiliations:** Pharmaceutics Research Group, School of Pharmacy, Institut Teknologi Bandung, Jalan Ganesha 10, Bandung 40132, Indonesia; yeyet@fa.itb.ac.id (Y.C.S.); sasanti@fa.itb.ac.id (S.T.D.)

**Keywords:** deoxyarbutin, nanostructured lipid carriers, nanoemulsion, cream, in vitro diffusion

## Abstract

The long-term use of topical hydroquinone as an anti-hyperpigmentation treatment has well-known, unwanted effects. Deoxyarbutin (4-[(tetrahydro-2H-pyran-2-yl)oxy]phenol) is a relatively new tyrosinase inhibitor, with stronger inhibitory potency than hydroquinone, that exhibited decreased cytotoxicity against melanocytes and other cells. This study developed novel nanostructured lipid carriers (NLCs) for improved topical delivery of deoxyarbutin (dArb), leading to improved depigmenting efficacy. dArb is a hydrophobic substance, but it easily degrades in aqueous medium and is thermolabile. Screening and optimisation of the solid lipid, liquid lipid, surfactant, co-surfactant and production methods were performed to choose the optimum particle size and stability for NLCs. One percent dArb NLCs were obtained from a combination of cetyl palmitate (CP) and caprylic/capric tryglicerides (Myr) in 12% total lipids using poloxamer 188 (P-188) and polyethylene glycol (PEG) 400 as a surfactant and co-surfactant, respectively, with a particle diameter of approximately 500 nm and a polydispersity index (PI) <0.4. These NLCs were produced using the simple method of high-shear homogenisation (10,000 rpm, 5 minutes) and ultrasonication (3.5 min). The compatibility between the substances in the formula was evaluated using Fourier Transform infrared spectroscopy (FTIR) and differential scanning calorimetry (DSC). The morphology of the NLCs was observed using transmission electron microscopy (TEM). In vitro penetration of dArb NLCs was evaluated and compared to the nanoemulsion (NE) and conventional emulsion (CR) delivery methods across Spangler’s membrane (SS). Delivery improvement was clearly observed, and after 8 h of application, dArb gel-NLCs showed the highest dArb penetration, followed by liquid NLCs, NE, and CR.

## 1. Introduction

Hydroquinone (HQ) is the main choice for hyperpigmentation therapy. However, the long-term use of HQ is known to cause several unwanted side effects including contact dermatitis, irritant dermatitis, post-inflammatory hyperpigmentation, ochronosis, and even depigmentation [1].

One of the promising HQ alternatives is deoxyarbutin (dArb) or 4-[(tetrahydro-2H-pyran-2-yl)oxy]phenol, which also works topically as an anti-hyperpigmentation agent by inhibiting tyrosinase in melanosomes of the epidermal melanocytes. dArb exhibited better inhibition properties and a safer profile than HQ because dArb is cytostatic, unlike the cytotoxic adverse effects of HQ [2].

**Figure 1:**
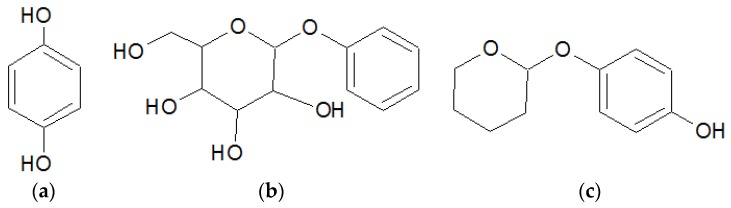
Molecular structure of (**a**) hydroquinone; (**b**) arbutin; and (**c**) deoxyarbutin.

dArb is a hydrophobic substance and is insoluble in water, but it easily degrades in aqueous conditions, as previously shown [3]. The chemical structures are shown in Figure 1. To increase the anti-hyperpigmentation activity of topical dArb, a potential candidate delivery system uses lipid-based nanoparticles. This system has been extensively studied for enhancing the percutaneous penetration of active substances and improving the oxidation stability of the aqueous formulation [4].

Nanostructured lipid carriers (NLCs) are one type of lipid nanoparticles that provide many advantages for the delivery of hydrophobic drugs such as dArb. Many studies have discussed the advantages of NLCs over other colloidal systems, including solid lipid nanoparticles (SLN), solid lipid microparticles, liposomes, nanoemulsions, and microemulsions [5,6]. NLCs are effective because the combination of solid and liquid lipid in NLCs produces lipid matrices that are less ordered than SLN, leading to decreased crystallinity and causing “imperfections” on the nanoparticles that act as a drug vehicle. Such a structure increases the loading capacity of the active compound and avoids/minimises the possibility of drug expulsion [7].

In this study, we developed NLCs using cetyl palmitate and caprylic/capric triglycerides as the lipid matrix. A low concentration of poloxamer 188 and polyethylene glycol (PEG) 400 acted as the surfactant and co-surfactant. The characteristics of the NLCs were evaluated. The NLC skin penetration profile was assessed and compared with other delivery systems to understand the mechanism of enhanced dArb permeation across the stratum corneum and full-thickness skin.

## 2. Materials and Methods

### 2.1. Materials

dArb was obtained from Asiavisions, Hongkong, China. Poloxamer 188, Cremophor RH-40, cetyl palmitate, myristyl myristate, dicaprylyl carbonate, and caprylic/capric triglycerides were surfactants and lipids purchased from BASF, Ludwigshaven, Germany. PEG-8-beeswax was purchased from Gattefosse, Paramus, NJ, USA. Whatman paper grade 1 was obtained from Whatman/GE Healthcare, Pittsburgh, PA, USA. Coconut oil was purchased from Henry Lamotte Oil Gmbh, Bremen Germany. Spermaceti wax and deionized water were obtained from Bratachem, Jakarta, Indonesia. Tween 80, palmitic acid, stearic acid, olive oil, squalene, cholesterol, oleic acid, linoleic acid, and liquid paraffin were purchased from Sigma-Aldrich, St. Louis, MT, USA.

### 2.2. Screening of Lipids and Surfactant

dArb and three solid lipids, cetyl palmitate (CP), myristyl myristate (MM), and PEG-8-beeswax (P8B), were evaluated for their melting points using a sublimation method. The solubility of dArb in each solid lipid was measured at each melting temperature by adding dArb incrementally into 1 g of the melted lipids with low speed magnetic stirring at 100 rpm.

The same procedure was applied to assess dArb solubility in two liquid lipids: caprylic/capric triglycerides (Myr) and dicaprylyl carbonate (CC), and in two surfactants: poloxamer 188 (P-188) and PEG-40-hydrogenated castor oil (CR-40).

### 2.3. Evaluation of Excipient Compatibilities

Melting points for the physical mixture of five different ratios of the chosen solid lipid (CP) and liquid lipid (Myr) were determined using a simple sublimation method. Afterwards, the solubility of dArb in each mixture was evaluated using the above procedure. The miscibility and solubility of each system was assessed for visual homogeneity upon melting and cooling.

To confirm the compatibility of excipients with the active substance, Fourier Transform Infrared (FTIR) spectrophotometry (Jasco 4200, Tokyo, Japan) was performed on dArb, CP, Myr, and P-188 alone, as well as mixtures of them. The samples were ground and mixed thoroughly with potassium bromide in a 1:5 (sample:KBr) ratio. The KBr discs were prepared by compression of the powder at 20 psi for three min on a KBr press. The spectra were scanned over a wavelength of 4500–500 cm^−1^.

### 2.4. Preparation of NLCs

The oil phase containing CP (9%) and Myr (2%) loaded with dArb (1%) was heated to 60 °C. The water phase containing P-188 (3%), PEG-400 (0.5%), sodium sulfite (0.5%), and deionised water was heated to 60 °C. The oil phase was added to the water phase, which was mixed with high shear (10,000 rpm, 5 min) at 60 °C. Particle size reduction was performed on the pre-emulsion using sonication probes (6500 J, 3.5 min), and the samples were directly cooled for 10 min to reach 25 °C.

### 2.5. Physicochemical Characterisation of Particles

Particle sizes and polydispersity indices for the NLCs were evaluated using dynamic light scattering (DLS), also known as photon correlation spectrometry (PCS), using a Beckman Coulter Delsa Nano C, (Indianapolis, IN, USA) at room temperature (25 °C). The samples were directly measured in disposable cuvettes.

To confirm dArb inclusion within NLCs, differential scanning calorimetry (DSC) measurements were performed using Linseis Thermal Analysis, Robinsville, NJ, USA. An NLC thermogram was compared with thermograms for each substance alone, blank NLCs, and dArb-loaded NLCs. The samples were heated from 25–100 °C at a heating rate of 2 K/min.

The microstructure of dArb-NLC particles was observed using a transmission electron microscope (TEM) (Hitachi H7500, Hitachi, Tokyo, Japan). The samples were diluted with double-distilled water and deposited on film-coated copper grids, and a droplet of phosphotungstic acid was added to dry overnight at room temperature. Dried samples were then evaluated visually using TEM.

### 2.6. Entrapment Efficiency (EE)

The amount of dArb entrapped in the NLCs was evaluated by an ultrafiltration method using Vivaspin filter tubes (Vivaspin, Goettingen, Germany) with a filter membrane that has a 3 kDa molecular weight cut-off. One millilitre of undiluted dArb-loaded NLCs was placed in the upper chamber of the tube, which was centrifuged at 13,000 rpm for 45 min (Minispin, Eppendorf, Hamburg, Germany). The filtrate was then diluted with methanol, and the amount of un-entrapped dArb was measured using validated high performance liquid chromatography (HPLC) (Waters 1525, Milford, MA, USA) with a reversed C-18 column, methanol:water (60:40) as the mobile phase, and a 280 nm detector. The EE was calculated with the following equation:

$$EE(\%)=\frac{\mathrm{Initial}\;\mathrm{dArb}-\mathrm{Unentrapped}\;\mathrm{dArb}}{\mathrm{Initial}\;\mathrm{dArb}}$$

### 2.7. High Performance Liquid Chromatography

dArb quantification was measured with a validated HPLC method using a Waters 1525 (Milford), equipped with a binary pump and a Waters 2487 UV/visible dual wavelength detector. The analytical column used was a Luna reversed C-18 (5 µm; 250 × 4.6 mm) Phenomenex (Torrence, CA, USA). The mobile phase consisted of methanol:water (60:40) at a flowing rate of 1 mL/min [3]. A run time of 6 min was established per sample, ensuring a dArb retention time of 4.5 min. Detection was performed at 280 nm, and 20 µL of each standard and sample were injected.

HPLC calibration of was determined in a dArb concentration range of 5–200 ppm in phosphate-buffered saline (PBS) pH 7.4 containing 5% Tween 80 for solubility. It resulted in a linear calibration curve with an *r*^2^ value of 0.9994. The limit of detection and limit of quantification were 1 ppm and 3 ppm, respectively.

### 2.8. In Vitro Penetration Studies

In vitro dArb penetration was performed using a Franz diffusion cell. The diffusion area was 4.52 cm^2^. This study compared penetration through a synthetic sebum membrane, which was prepared by impregnating Whatman paper grade 1 with Spangler’s synthetic sebum for 12 h. Whatman paper grade 1 is a 9-cm diameter cellulose filter with a pore diameter of 11 µm. The composition of Spangler’s sebum is shown in Table 1.

A total of 500 mg of sample containing 5 mg dArb was applied to the membrane. The receptor fluid was PBS pH 7.5 containing 2% Tween 80 in a volume of 50 mL. The amount of dArb penetration was measured after 15 min, 30 min, 1 h, 2 h, 4 h, 6 h, and 8 h, respectively, using the HPLC method mentioned above.

To understand the difference in penetration profiles based on delivery system, diffusion tests were performed for 4 formulations: NLC, NLC-gel, NE, and conventional cream emulsion (CR), all containing 1% dArb.

### 2.9. Statistics

All experiments were performed in triplicate in independent experiments. Data are expressed as the mean ± standard deviation in tables or with error bars in the figures.

The significant differences for the in vitro penetration studies were evaluated using two-factor analysis of variance (ANOVA) with replication at a probability level of 0.05. The calculations were performed using Microsoft Excel (Microsoft Corp., Redmond, WA, USA).

## 3. Results and Discussion

### 3.1. Screening and Formulation of NLCs

The solid lipids CP, MM, and P8B showed melting points of 51.37 ± 1.06 °C, 42.57 ± 0.85 °C, and 69.80 ± 1.10 °C, respectively. The solubility of dArb in each of the solid lipids was 81.42 ± 0.58 mg/g (at 55 °C), 85.36 ± 3.31 mg/g (at 47 °C), and 149.71 ± 3.28 mg/g (at 75 °C), respectively. In liquid lipids, dArb showed an increased solubility of 105.37 ± 2.94 mg/g in Myr compared to 71.64 ± 2.63 mg/g in CC. Therefore, Myr was chosen as the liquid lipid. CP, the solid lipid with the highest solubility, was selected to maximise loading. The moderate melting point of CP was a positive factor. At room temperature, the lipid nanoparticles stay in a solid state, whereas during and after penetration across the stratum corneum, the CP matrix releases dArb from the NLCs with a good profile.

dArb solubility was also evaluated in two surfactants, P-188 and PEG-40 hydrogenated castor oil (Cremophor RH-40: C-40). The values were 1.41 ± 0.04 mg/g in 1% P-188 aqueous solution and 1.58 ± 0.08 mg/g in 1% C-40 aqueous solution. When choosing the appropriate surfactant, the one with lower solubility is preferred because the surfactant should act as a physicochemical barrier between the lipid matrix containing dArb and the outer water phase. The high solubility of dArb within the surfactant will stimulate the partitioning of dArb from the lipid matrix, resulting in some of the dArb solubilised in the surfactant. Thus, the main purpose for including dArb within the lipid matrix was not achieved.

The solid and liquid lipids chosen (CP and Myr) were physically mixed in several different compositions and were evaluated for their melting points and miscibilities. Visually, all physical mixtures demonstrated good miscibility of the two lipids. The melting points of the four mixtures containing more CP than Myr were only slightly different from the melting point of CP alone, ranging from 50.0–51.8 °C as shown in Table 2, whereas the mixture that contained more Myr than CP was semisolid or waxy at room temperature. When combined with dArb, the data (Table 3) indicated that a ratio of CP:Myr 8:2 exhibited ideal solubility at 150.23 ± 2.34 mg/g and used a small amount of liquid lipid. These results agree with Attama et al., who found that lipid matrices with a certain degree of “imperfection“ were ideal for high-loading NLC formulations [8].

**Figure 2:**
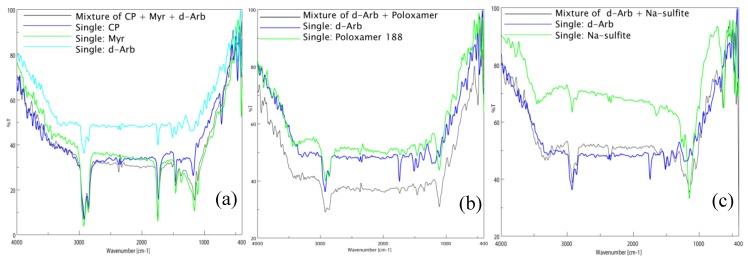
Fourier transform infrared spectra of (**a**) CP, Myr, and dArb alone and a mixture of CP-Myr-dArb; (**b**) dArb and P-188 alone and a mixture of dArb-P-188; (**c**) dArb and sodium sulfite (NS) alone and a mixture of dArb-NS. dArb: deoxyarbutin; CP: cetyl palmitate; Myr: caprylic/capric triglycerides; P-188: poloxamer 188.

The FTIR spectra of single substances and their physical mixtures confirmed that CP, Myr, and dArb were compatible and that dArb was compatible with the surfactant (P-188) and the antioxidant (sodium sulfite), as shown in Figure 2. There were no new peaks observed in the spectra of any of the mixtures, indicating that there were no molecular interactions between the substances.

**Figure 3:**
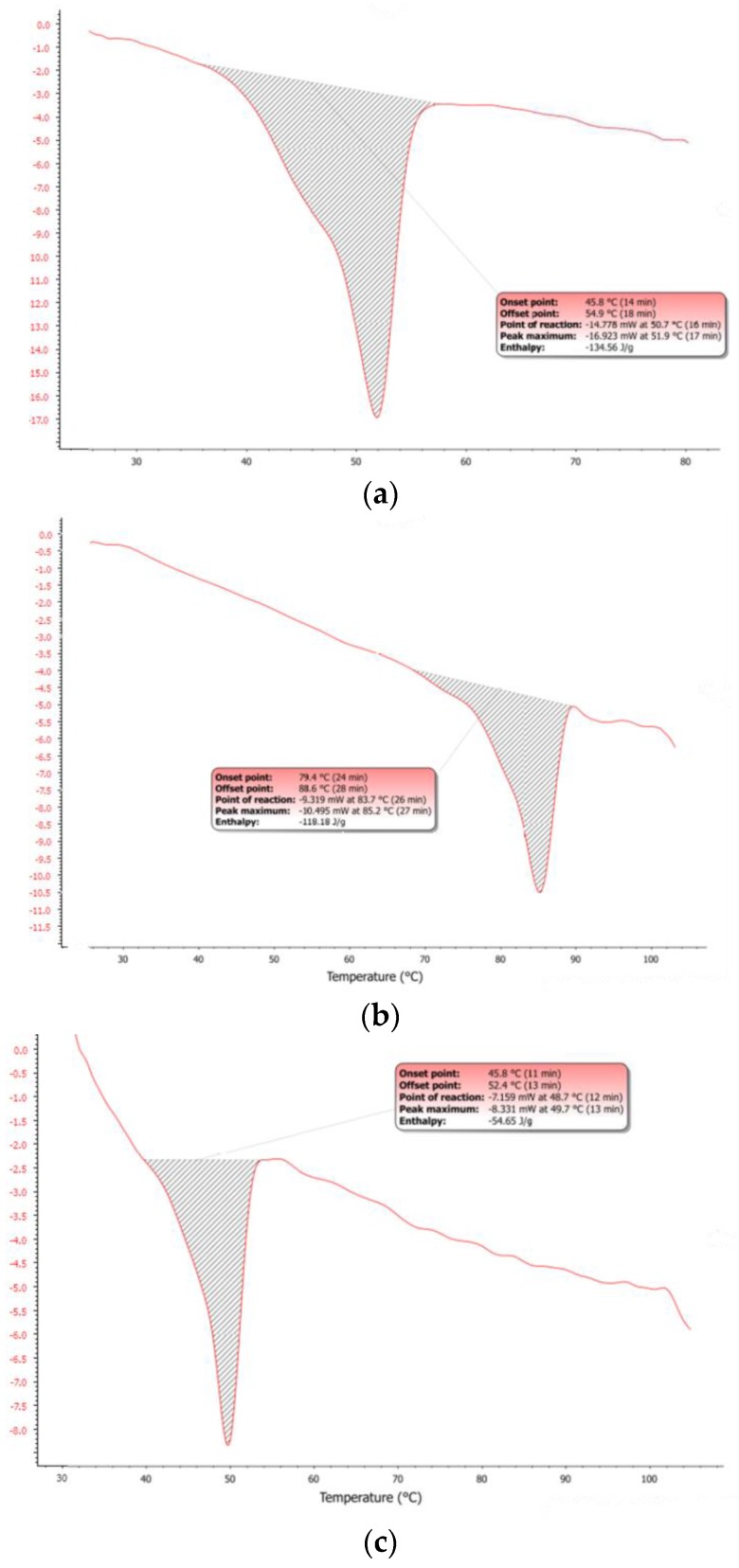

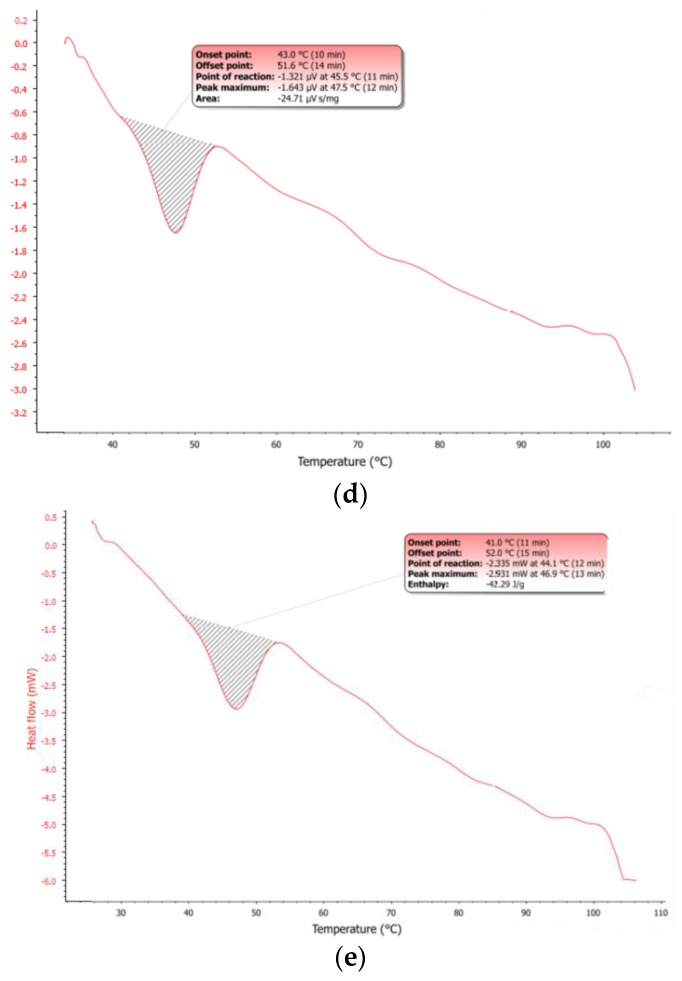
Differential scanning calorimetry (DSC) thermograms of (**a**) bulk CP; (**b**) bulk dArb; (**c**) physical mixture of dArb, CP, and Myr; (**d**) blank-NLC; and (**e**) dArb-NLC. NLC: nanostructured lipid carriers.

The observed dArb-lipid compatibilities and the inclusion of dArb within lipid matrices were explained by the differential scanning calorimetry (DSC) thermograms shown in Figure 3a–c. The DSC for bulk CP showed an endothermic curve ranging from 45.8–54.9 °C, with a peak melting point of 51.9 °C. However, the dArb thermogram began to form an endothermic curve at 79.4 °C that ended at 88.6 °C and peaked at 85.2 °C. After dArb was solubilised in a mixture of lipids (CP and Myr) with the ratio indicated in Table 4, the DSC thermogram showed an endothermic curve only at 45.8–52.4 °C and did not exhibit a curve near the dArb melting point (80–90 °C). This result indicated that dArb was completely dissolved in the lipid mixture and that the inclusion of dArb within the lipid matrix was possible.

The mixture of solid lipid (CP), liquid lipid (Myr), surfactant (P-188), co-surfactant (PEG 400), antioxidant (sodium sulfite), and dArb was optimised. The optimised formula for 1% dArb NLCs is shown in Table 4 and describes a stable smallest particle size NLC (434.9 ± 21.48 nm) with a polydispersity index of 0.219 ± 0.004. Optimisation of the preparation technique yielded a simple production method using high shear mixing (Ultrathurrax T25, Ika, Germany) at 10,000 rpm for 5 min and 60 °C followed by size reduction using sonophoresis (Misonix Sonicator, Farmingdale, NY, USA) at 6500 J for 3 min 30 s.

A mixture of dArb and the lipid phase was heated to 60 °C. The solution was clear, and no crystals were detected, indicating complete dArb solubilisation in the lipid matrix and agreeing with the DSC thermograms in Figure 3. A two-step NLC production method using high shear mixing coupled with ultrasonication was expected to result in increased homogeneity and reduced nanoparticle dispersion [9]. These advantages were clear from the polydispersity index of less than 0.25, which indicates uniformity of the hydrodynamic lipid particle diameter and a minimal aggregation tendency [10]. Particle sizes of less than 500 nm were successfully obtained, which is an ideal value for z [11].

Figure 3d,e shows that the DSC dArb-NLC thermogram exhibited a similar profile to blank NLCs and did not have a peak at the dArb melting point. This finding confirmed that dArb was successfully included in the lipid matrix. Further analysis comparing the physical mixture of CP-dArb-Myr (Figure 3c) and dArb NLC (Figure 3e), presented in Table 5, showed that the NLC endotherm curve broadened (11.0 °C breadth) compared to the physical mixture (6.8 °C breadth), and shifted to a slightly lower temperature, from 49.7 °C to 46.9 °C. Other than a possible effect from adsorbed emulsifier molecules, this phenomenon also conformed to the expected behaviour of smaller particle sized nanodispersed materials [12] according to the Gibbs-Thomson equation [13]:

$$\ln\frac{T}{T_{0}}=\;-\frac{2\gamma_{\mathrm{sl}}V_{s}}{r\Delta H_{\mathrm{fus}}}$$

in which *T* is the melting temperature of a particle with radius *r*, *T_0_* is the melting temperature of the bulk material at the same external pressure, *γ_sl_* is the interfacial tension at the solid-liquid interface, *V_s_* is the specific volume of the solid, and *ΔH_fus_* is the specific heat of fusion.

The equation demonstrates that smaller nanoparticles would have a lower melting temperature. The endothermic curve broadening observed was due to the polydispersity of nanoparticles, suggesting the presence of different sizes of nanoparticles that melted at different temperatures [8]. Decreased NLC enthalpy indicated a decrease in the degree of crystallinity, which was an expected advantage of the NLC system [14].

**Figure 4:**
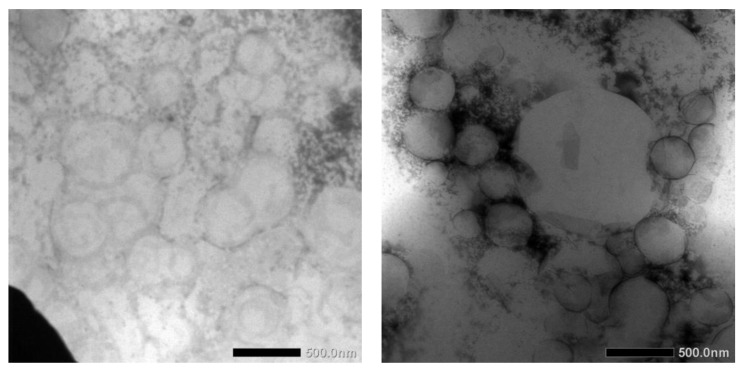
Transmission electron microscopy imaging of (**left**) blank NLCs and (**right**) dArb-NLCs.

TEM images of blank NLCs and dArb-NLCs are shown in Figure 4 and show spherically shaped particles with sizes of approximately 400–500 nm, which generally agrees with previously reported photon correlation spectroscopy (PCS) results. The differences observed between the images were primarily caused by different positions and amplification factors. Moreover, different sample preparations and the different principles behind PCS and TEM might introduce slight variations. PCS detects light scattering effects that are used to calculate average particle size, whereas TEM can “visually” measure the particle dimension and allow observation of their actual shapes [15].

Triplicate measurements of EE resulted in a convincing 99.821% ± 0.004%. This result was possibly due to only 1% of the dArb being loaded into a total of 12% lipids, while the maximum solubility of dArb was approximately 1 in 8 parts of mixed lipids. An ultrafiltration method was used to evaluate the EE of dArb in the NLC and was able to quickly determine the amount of unentrapped dArb. A smaller cut-off molecular weight (MW) value of the filter provided more accurate results because the filtrate separated by centrifugation was limited to only the soluble dArb in the water + surfactant system, as long as the MW of the substance was much smaller than the cut-off value.

### 3.2. In Vitro Skin Penetration Studies

Table 6 shows the formula used for skin penetration studies. All formulations contained 1% dArb with the same percentage of lipid phase and surfactant. Such formulas were designed to minimise the influence of ingredient composition, thereby evaluating only responses due to differences in the delivery system.

This study compared dArb-NLC in its original liquid form and a hydroxypropyl methylcellulose (HPMC)-thickened formulation. Higher topical formulation viscosity might form a film layer; however, due to the resulting occlusive effect, it enhances the dermal penetration of the active substance. Skin penetration profiles for a dArb nanoemulsion (NE) and a conventional 1% dArb cream emulsion (CR) were evaluated against NLCs. NE is a colloidal dispersion containing only liquid lipid (Myr) as the lipid phase, and CR is a semisolid emulsion containing micron-sized globules of dArb-loaded lipid and wax.

The membrane used to represent the penetration barrier of skin was a synthetic sebum membrane consisting of Whatman paper impregnated with a mixture of several lipids modelled after the semi-polar lipids found in the epidermis extracellular matrix and known as Spangler’s synthetic sebum [16]. Spangler’s sebum was chosen instead of the mixture proposed by Wertz [17] because Spangler suggested a more complex lipid/oil composition that better mimics actual physiologic sebum. Spangler’s membrane (SP) represents the barrier properties of the stratum corneum without the appendages.

**Figure 5:**
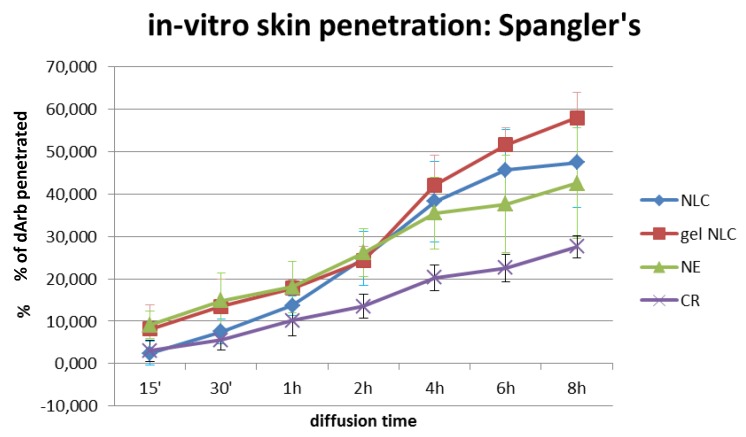
Amount (%) of in vitro skin penetration of dArb in NLC, gel-NLC, NE, and CR through Spangler’s membrane. Nanoemulsion (NE); cream emulsion (CR).

As expected, the dArb penetration through the SP for different delivery systems (Figure 5) showed that gel-NLCs generated the highest dArb penetration (57.97% ± 6.08%) after 8 h of topical application, followed by NLCs (47.39% ± 10.59%), NE (42.49% ± 13.07%), and CR (27.58% ± 2.59%). The in vitro diffusion profiles demonstrated that both NLCs and gel-NLCs facilitated gradual and improved permeation of skin lipids and confirming the effectiveness of these systems at increasing the intercellular permeation of dArb.

A two-way ANOVA of the data showed a *p* value of 2.2 × 10^−10^ (Table 7), indicating a significant difference in dArb penetration between the four delivery systems. Further analysis with the least significant difference (LSD) method was performed to understand which delivery system showed a significant difference in dArb penetration compared to gel-NLCs. The LSD value was 6.705, and the absolute values of the mean differences of the two groups were calculated. They were 5.084, 4.503 and 16.095 for gel-NLCs vs. NLCs, gel-NLCs vs. NE, and gel-NLCs vs. CR, respectively. This result suggested that there was a significant difference between gel-NLCs and CR in terms of dArb penetration. However, although NLCs and NE did not show significant differences in the amount of dArb penetration, their penetration profiles over time were observably different.

The permeation profiles of gel-NLCs were slightly higher than that of NLCs and might indicate that the gelling agent HPMC played a role enhancing penetration. Cellulose derivatives are known to have film-forming properties that reduce trans epidermal water loss (TEWL) and cause skin hydration, increasing the percutaneous absorption of lipid nanoparticles [18].

The permeation of NE across the SP before 2 h of application was similar to gel-NLCs and was improved over liquid NLCs because the liquid oil NE globules were more fluid than the solidified lipid matrices of the NLCs. The fluidity, and thus elasticity, of the nanoglobules resulted in a quicker permeation rate using an intercellular route across the brick-and-mortar-like model of the stratum corneum represented by the SP. However, after the 2nd hour, the “imperfect” lipid matrices of the NLCs started to release more dArb to the receptor solution, surpassing NE. The solid nature of the NLC lipid matrix caused the entrapped dArb to be released more slowly from the matrix and into the receptor fluid than the solubilised dArb within the liquid oil NE globules.

As predicted, the CR showed the least dArb permeation into the receptor medium. A conventional cream formulation generally has much larger globule sizes and is less permeable across the stratum corneum lipids, consistently showing the lowest concentration of dArb in the receptor medium.

## 4. Conclusions

An NLC formulation containing dArb using a simple method, few lipids, and a low surfactant concentration was developed. dArb skin permeation can be successfully increased using the active NLC delivery system. The addition of HPMC to the NLC formulation as thickener and gelling agent showed an additional benefit as a penetration enhancer. Therefore, this delivery system provides opportunity to increase the efficacy of dArb to inhibit tyrosinase during melanogenesis. These results indicated that the dArb gel-NLC formulation might be a promising candidate in the search for an effective substitute for topical HQ.

## Figures and Tables

**Table 1:** Composition of Spangler’s synthetic sebum [12].

	% w/w
Palmitic acid	10.0
Stearic acid	5.0
Coconut oil	15.0
Liquid paraffin	10.0
Spermaceti	15.0
Olive oil	20.0
Squalene	5.0
Cholesterol	5.0
Oleic acid	10.0
Linoleic acid	5.0

**Table 2:** Melting points of several solid lipids and mixtures of CP and Myr (°C).

Melting Point ^a^	
Solid lipids	
CP	51.37 ± 1.06
MM	42.57 ± 0.85
P8B	69.80 ± 1.10
Physical mixture of CP:Myr	
9:1	51.77 ± 0.70
8:2	50.60 ± 0.80
7:3	51.20 ± 0.75
5:5	50.03 ± 0.85
3:7	semisolid/waxy at RT

^a^ mean of 3 runs. CP: cetyl palmitate; Myr: caprylic/capric triglycerides; P8B: polyethylene glycol 8-beeswax; RT: room temperature.

**Table 3:** Solubility of deoxyarbutin (dArb) in several solid lipids, liquid lipids, a mixture of CP:Myr, and surfactants (mg/g).

dArb Solubility ^a^ (mg/g)	
Solid lipids	
CP	81.42 ± 0.58
MM	85.39 ± 3.31
P8B	149.71 ± 3.29
Liquid lipids	
Myr	105.37 ± 2.94
CC	71.46 ± 2.63
Physical mixture of CP:Myr (at 55 °C)	
9:1	89.82 ± 3.41
8:2	150.23 ± 2.34
7:3	160.97 ± 2.13
5:5	193.92 ± 3.12
Surfactant (1% aqueous solution)	
P-188	1.41 ± 0.04
C-40	1.58 ± 0.08

^a^ mean of 3 runs. MM: myristyl myristate; CC: dicaprylyl carbonate; P-188: poloxamer 188; C-40: PEG-40 hydrogenated castor oil.

**Table 4:** Formula of dArb nanostructured lipid carriers (NLCs).

		% w/w
**Active substance**		
Deoxyarbutin	dArb	1
**Lipid phase**		
Cetyl Palmitate	CP	9
Caprylic/Capric Triglycerides	Myr	2
**Water phase**		
Poloxamer 188	P-188	3
Polyethylene glycol 400	PEG	0.5
Sodium sulfite	NS	0.5
Deionized water		ad. 100

**Table 5:** Thermal properties of the physical mixture and dArb NLCs.

Formula	Onset Temperature (°C)	Peak Temperature (°C)	Offset Temperature (°C)	Offset–Onset Temperature (°C)	Enthalpy (J/g)
Physical mixture	45.8	49.7	52.4	6.8	−54.65
dArb NLC	41.0	46.9	52.0	11.0	−42.29

**Table 6:** Formulas for skin penetration studies.

% w/w		NLC	Gel-NLC	Nano-emulsion (NE)	Conventional Cream (CR)
					
**Active substance**					
Deoxyarbutin	dArb	1	1	1	1
					
**Lipid phase**					
Cetyl Palmitate	CP	9	9	-	-
Caprylic/Capric Triglycerides	Myr	2	2	11	2
Cetostearyl Alcohol	CS	-	-	-	12
					
**Water phase**					
Hydroxypropyl methylcellulose	HPMC	-	4	-	-
PEG-40 hydrogenated castor oil	C-40	-	-	-	3
Poloxamer 188	P-188	3	3	3	-
PEG 400	PEG	0.5	0.5	0.5	-
Sodium Sulfite	NS	0.5	0.5	0.5	0.5
Deionized water		84	84	84	81.5

**Table 7:** Two-way analysis of variance calculation of the in vitro penetration study.

Source of Variation	SS	df	MS	F	*p* Value	F crit
Sample	2946.006	3	982.002	24.961	2.199 × 10^−10^	2.769
Columns	16047.624	6	2674.604	67.986	6.603 × 10^−24^	2.265
Interaction	1456.696	18	80.927	2.057	0.021	1.791
Within	2203.061	56	39.340			
Total	22653.388	83				

SS: sum square, df: degree of freedom, MS: mean square.
